# Overdamping of vibration resonances by liquid crystal elastomers

**DOI:** 10.1038/s41598-024-76952-3

**Published:** 2024-10-28

**Authors:** Waiel Elmadih, Andrew Terentjev, Hsin-Ling Liang, Eugene Terentjev

**Affiliations:** 1 Metamaterials Ltd, The Ingenuity Lab, Jubilee Campus, Nottingham, NG7 2TU UK; 2 Cambridge Smart Plastics Ltd, 18 Hurrell Road, Cambridge, CB4 3RH UK; 3 Cavendish Laboratory, University of Cambridge, JJ Thomson Avenue, Cambridge, CB3 0HE UK

**Keywords:** Vibration, Resonance, Damping, Liquid crystal elastomers, Mechanical engineering, Polymers

## Abstract

This work aims to compare the capability of vibration attenuation by standard elastomeric polymers, and by the new anomalously damping nematic liquid crystal elastomer. We use the most mainstream materials in both categories, and design two testing platforms: the ASTM-standard constrained layer plate resonance geometry, and the attenuation of resonances in a commercial device (electric drill) where the damping polymers were inserted into the casing. In the standard plate resonance testing, we find that LCE outperforms all standard damping materials, moreover, it brings the vibrating plate into the overdamped condition, which is unique for a non-fluid dissipative system. In the attenuation of high-frequency vibrations of a device, we also found LCE dissipates these vibrations much better, although we did not find the optimal insertion configuration for the damping polymer, and did not reach overdamping.

## Introduction

Liquid Crystalline Elastomers (LCE) are thermosets that belong to the family of “smart plastics”. They combine the softness and elasticity of elastomers, with the orientational order properties of liquid crystals, resulting in the ability for these elastomers to undergo large reversible spontaneous deformation when subjected to external stimuli. This unique ability to actuate and perform work without mechanical parts has made LCE potentially attractive for applications in fields such as soft robotics, sensors and tissue engineering, and has driven their research for over 30 years^1^. In contrast, the second unique property of LCE called “soft elasticity”, which is also the direct consequence of internal orientational order in the elastic network, has never found a suitable application concept. Yet, in recent years, there has been a renewed focus and attention to this aspect of LCE and several discoveries made (notably, the anomalous damping in LCE^2,3^ and the pressure-sensitive adhesion in LCE^4,5^). This made LCE into a much more realistic application concept, compared with the actuation route.

These recent advances and findings in the field of LCE damping and adhesion have also brought to focus a conceptual misunderstanding that occurred in the original work of 2001^2,6,7^ when these effects were associated with ‘dynamic soft elasticity’. On the one hand, it was natural to expect that when the soft elastic stress-strain plateau occurs, where the effective modulus is very low or zero, that would lead to a high loss factor and seen as the enhanced dissipation. However, soft elasticity is a fundamentally equilibrium effect, relating the local tension in the network chains to the (re)orientation of local nematic director. Recently, it has become clear that the enhanced damping and its associated pressure sensitive adhesion are much stronger at high frequencies. Especially after the so-called Master Curves of the linear dynamic mechanical response have been constructed and examined^8,9^, it was found that the loss factor reached its maximum at frequencies of 10 kHz or even more. No ‘soft elasticity’ could occurs at such rates of strain.

It is clear now that the enhanced LCE damping relies on the rotational viscosity of the nematic director (and its coupling to the network elasticity in LCE), and this is a fundamentally non-equilibrium high-frequency phenomenon with many analogies with the rotational viscosity of liquid crystals, which in turn is based on the pair correlations of interacting anisotropic mesogens^10,11^. While the consistent theoretical understanding of these effects is being developed, here we undertake an additional experimental work to focus on the high-frequency aspects of vibration damping in LCE.

The state-of-the-art in vibration control technologies covers a broad range of topics, such as ‘passive vibration control’, which includes a variety of damping materials, tuned mass dampers and base isolators, and also exciting novel ideas of ‘acoustic black holes’^12,13^ and ‘band-gap’ phononic systems^14,15^. The ‘active vibration control’ methods include a variety of actuators and feedback systems, while various ‘autonomous damping systems’ are based on self-tuning controllers or energy harvesting methods. Advanced damping materials such as LCE belong to the ‘passive’ range, although the ability to change the liquid-crystalline order in LCE (and thus switch the damping on and off at will) could place them into the ‘semi-active’ smart materials category.

We carry out experiments on LCE vibration damping in two distinct settings: monitoring vibration resonances in a ‘standard’ metal plate (to have the fundamental and experimentally clean data on vibration dissipation), and monitoring vibration of a ‘model device’ (a commercial handheld drill, in our case) to assess how the effect of introducing damping elastomer layers affects vibration resonances in such a device as a whole. We compare two elastomeric dampers: the basic LCE, and the standard silicone rubber with a similar crosslinking density, to ascertain the role of liquid-crystallinity in the damping increase.

## Plate vibration

**Fig. 1:**
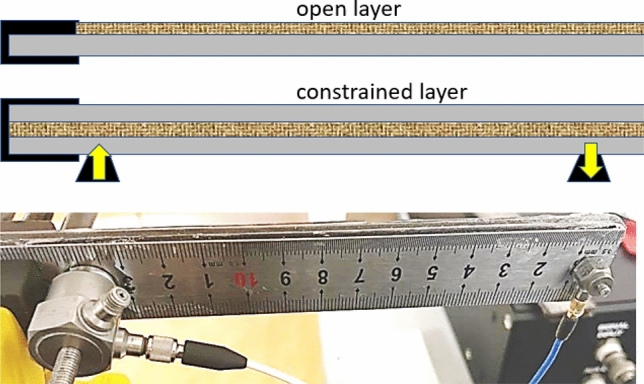
The illustrations of constrained layer geometry of vibrating plate damping, and the positioning of actuator/detector on the metal side. The use of metal ruler as plates helps maintaining the same position of actuator/detector and thus exploring the same normal modes of the construction in all tests.

The ASTM E756 standard for the testing of plate vibrations specifically focuses on the contrast between ‘open layer’ and the ‘constrained layer’ damping. With a metal plate vibrating, the great difference in the mechanical impedance between it and a soft elastomer makes the open layer damping inefficient. The constrained layer damping has the vibrating metal plates of both sides of the elastomer, and thus forces the mechanical deformation into the dissipative matrix, enhancing damping. See an extended discussion of this issue in the 1996 Tomlinson report^16^. We, therefore, only examine the ‘sandwich specimen’ in the ASTM E756 language, where the dissipating elastomer layer is constrained between two identical metal plates, with the vibrating actuator and the detecting accelerometer are attached as illustrated in Fig. 1.

As mentioned in Introduction, we compare the vibration of the bare metal plate with that damped by the 2 mm-layer of 10%-crosslinked silicone elastomer, with that damped by the 2 mm-layer of the 10%-crosslinked nematic LCE in polydomain state. We know from past studies that polydomain nematic LCE is a better damper than the aligned monodomain one, but here we wanted to compare the role of elastomer genesis: there has been a lot of discussion and discovery about the difference between the nematic LCE being crosslinked in its isotropic phase and then cooled (making this ‘isotropic genesis’) and the one crosslinked in the non-aligned (polydomain) nematic state, making this ‘nematic genesis’^17–19^. The isotropic genesis allows the least amount of internal constraints in the nematic elastomer network, and their polydomain state is the most natural ‘spin-glass’ texture^17,20^, while crosslinking in the nematic phase retains the internal topology of the Schlieren texture of the liquid nematic, which restricts domain wall motion. Hence, in all results, we compare the bare-metal, the silicone, the nematic- and the isotropic-genesis LCE of the same crosslinking density.

For completeness, we also test the vibration damping with a 2 mm-layer of Sorbothane, one of the industry-leading damping materials invented in 1982, based on hydrogen-bonded polyurethane and having the enhanced damping due to a high fraction of dangling chains in the matrix^21^. It is not covalently crosslinked, and so the comparison with silicone and LCE is not completely fair (a ‘viscous fluid’ would have a much higher dissipation than an elastomeric network), but we nevertheless present its results to better assess where the LCE are on the spectrum of ‘best dampers’ available today.

**Fig. 2:**
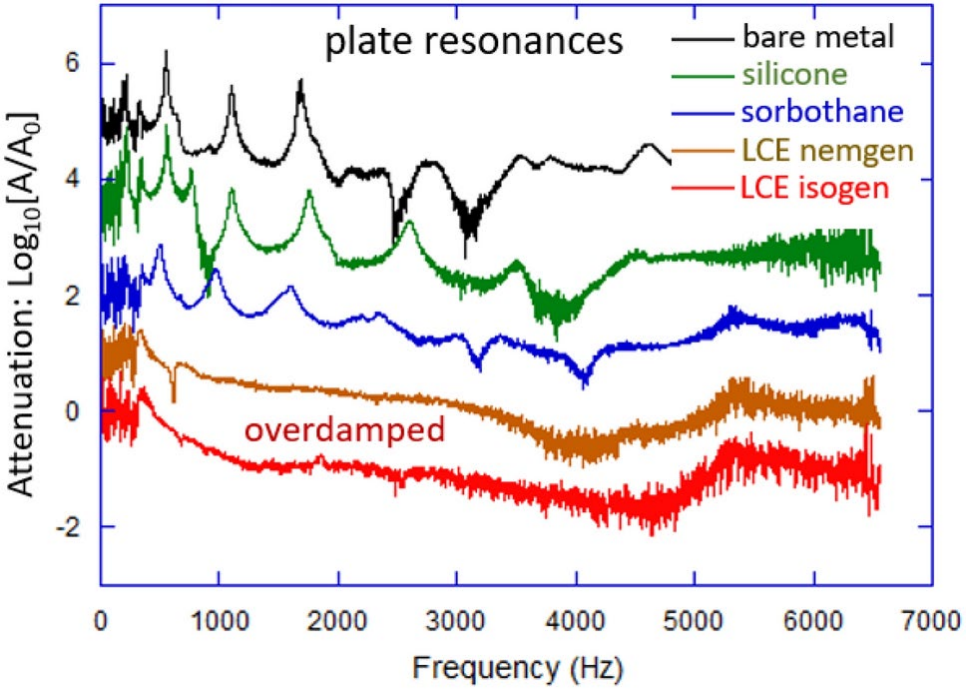
The full spectrum of plate vibration attenuation (FFT of the raw data on position v. time) of the bare metal plate, and the four 2 mm-damping systems in the identical constrained layer geometry. The low-frequency edge of this spectrum is noise because the FFT of a limited-size sample; the curves are artificially shifted upwards to avoid overlapping their key features.

Figure 2 illustrates the resonances in the plate in the specified geometry. The curves are all coming to (0,0) on this plot, but only the red curve for the isotropic-genesis LCE is kept as original: the other curves are artificially lifted upwards in the plot to enable the reader to better distinguish their features. In our setup, we use a piezo-actuator to induce mechanical vibrations at frequencies above 100 Hz. We do not send low-frequency signals because the limited size of our samples makes the low-frequency oscillations very noisy after the Fourier Transformation of raw data, and our targeted resonance frequencies of the samples are well above this limit. Consequently, the results displayed contain noise below 300 Hz. The bare metal plate shows five clear consecutive plate resonances (normal modes in this geometry), with the two above 2 kHz having their phase inverted.

**Fig. 3:**
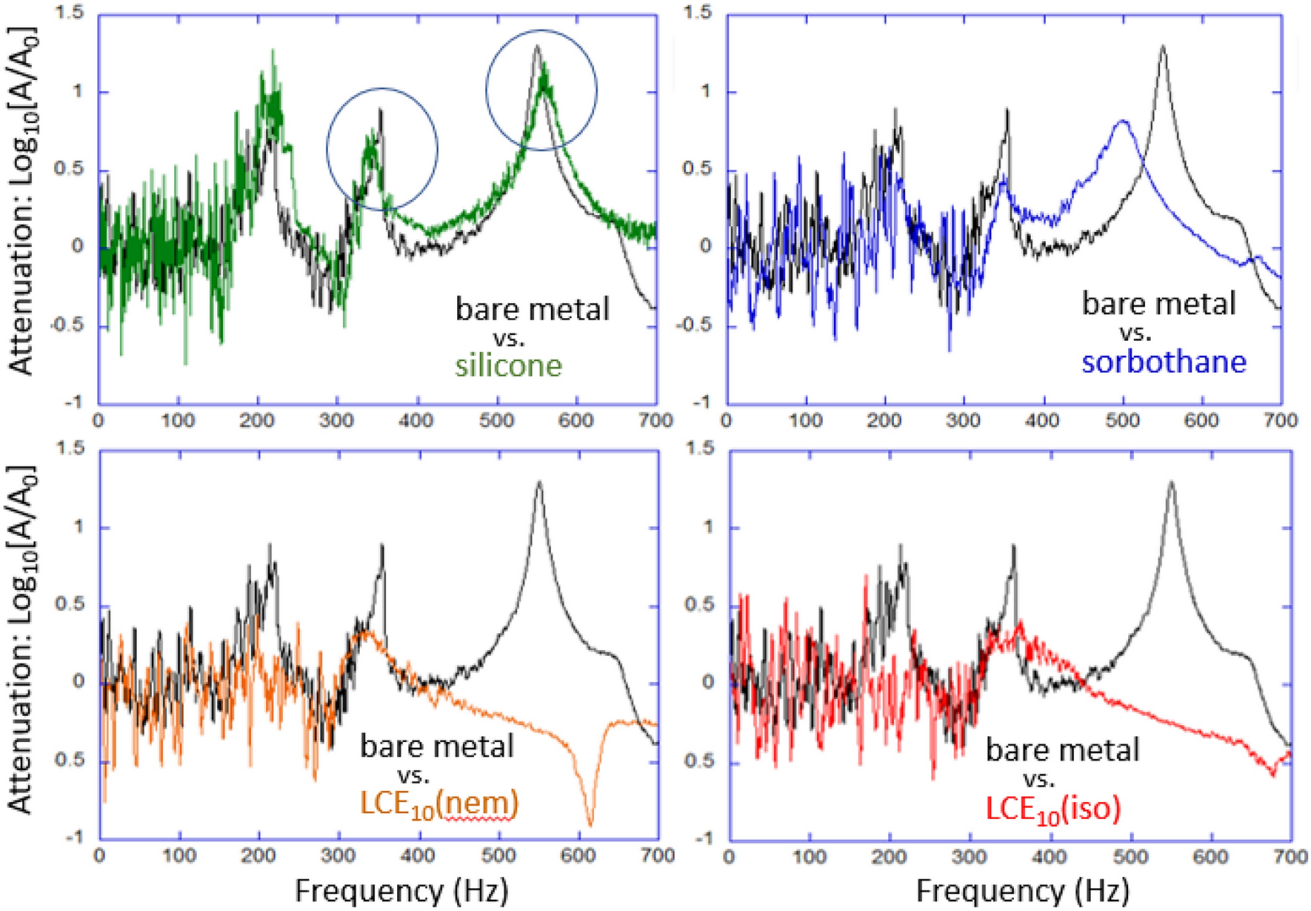
The resonances at 350 Hz and at 550 Hz (circled in the first plot) are examined, comparing the effect of the four damping materials.

It is clear that both silicone and Sorbothane do an increasing good job in damping these normal modes, even though a 1–1 matching of resonance peaks is not always perfect: the mechanical systems are slightly different and normal modes shift slightly, both in their principal frequency and their phase. It is also clear that both LCE dampers essentially eliminate all high-frequency plate resonances. Figure 3 zooms in, and focuses on a narrow frequency range, examining the two first resonances: at 350 Hz and at 550 Hz. We can see that a pure elastomeric silicone does achieve some damping, while largely preserving the resonance spectrum. In contrast, Sorbothane layer shifts the normal mode for the second peak to a lower frequency. The two LCE layers achieve a very significant damping of the first normal mode, but the nature of the second resonance is changed completely. Here the attenuation spectra curves are not shifted, so we see that the attenuation above 400 Hz is consistently negative. Hence we cannot interpret the ‘feature’ at 630–660 Hz as any kind of resonance shifted upwards from the bare 550 Hz: instead we believe it is a reflection of some other process seen as the 650 Hz ‘shoulder’ in the bare metal plate vibration, while the 550 Hz normal mode is damped completely by both LCEs. The reason why different vibration modes have different nature of their damping by LCE has been theoretically discussed in 2002, finding that the shear (S) acoustic waves dissipate much stronger, while the longitudinal (P) waves behave no differently than in an ordinary elastic network^22^: different normal modes would have a different distribution between S/P waves.

**Table 1:** Comparison of *Q*-factors for the two resonances, calculated from the data presented in Fig. 3.

	Bare metal	Silicone	Sorbothane	LCE (nem)	LCE (iso)
@350Hz	13 ± 2	9 ± 3	9 ± 4	4 ± 2	3 ± 1
@550Hz	13 ± 2	8 ± 2	6 ± 2	0	0

Each constrained layer system underwent 20–30 independent tests, with each experiment comprising 16,000 data points between zero and 6500 Hz for analysis, .Table 1 summarizes this resonance analysis by presenting the *Q*-factors calculated for all the data plotted in Fig. 3 (the *Q*-factor is defined as $f\textrm{peak}/\Delta f_\textrm{FWHM}$ and its significance lies in its direct connection with the loss factor of dynamic-mechanical analysis: $\tan \delta = 1/Q$). We are compelled to trust the data and the calculation of the 550 Hz resonance more, because the 350 Hz one is too close, and possibly affected by the growing noisiness at low frequencies. Therefore, we reach a conclusion that both LCE damping layers achieve the fully ‘overdamped’ condition, which is unique for an elastomeric damper material. The full frequency-range plot in Fig. 2 makes this conclusion even more clear than the *Q*-factor calculation. Remarkably, in this damping geometry, the industry-leading Sorbothane is not delivering even a comparable dissipation to the polydomain nematic LCE layer.

## Device vibration

**Fig. 4:**
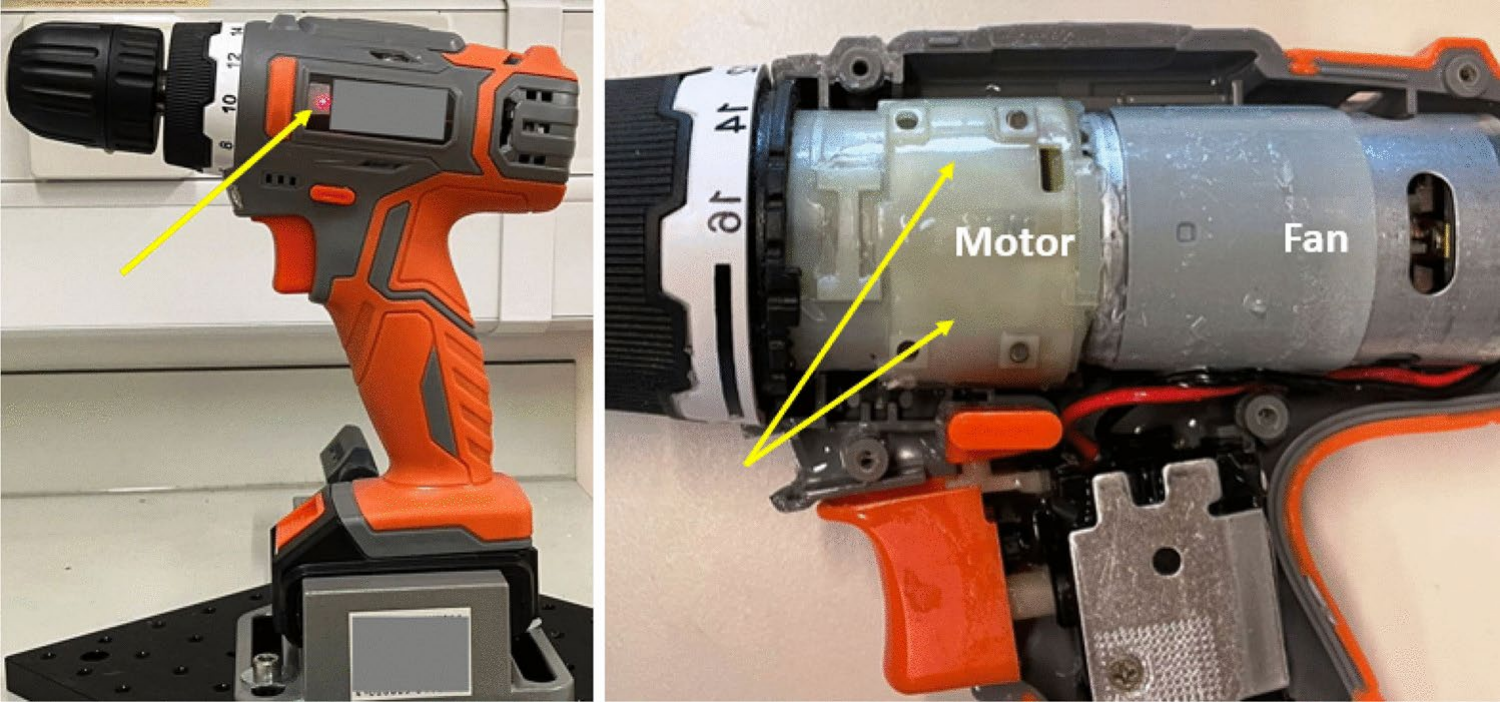
The illustrations of our device attenuation setup. The drill is mounted in a heavy vice and the vibration of its body monitored by the laser Doppler vibrometer reflecting the beam from a fixed spot, as marked in the photo. We insert a 2 mm damping elastomer into the gap between the motor and the outer body shell, as marked in the photo.

In the study of vibration damping of an actual ‘model device’, we made some choices. Obviously, it was not possible to cover a great variability of such devices—from the dental drill, to the computer motherboard fan, to building equipment and vehicles. In all of these, there is a great need to dissipate the vibration energy for performance, environmental and health-related reasons. Understanding that, we chose the simplest and most familiar device (a handheld drill) and a particular (not necessarily optimal) positioning of the damping layer inside, merely aiming at the comparison of LCE and ordinary elastomer damper in identical operating conditions. Figure 4 illustrates the device and the experiment geometry. The drill was operated at the maximum zero-load speed of 650 rpm (which translates to the cyclic frequency of 10.8 Hz).

For practical reasons, we were not able to insert Sorbothane into the ‘damping cavity’: that would require a dedicated device assembly step. We only used the isotropic-genesis LCE, having learned from the plate resonance study that it is similar but better than the nematic-genesis LCE. So, in this experiment we only compared the ‘bare’ device, with silicone and isotropic-genesis LCE damping pads inserted into the cavity surrounding the motor, see Fig. 4. We fill this gap with a pre-crosslinked viscous polymer and let the elastomer be crosslinked in-situ, before closing the device casing.

**Fig. 5:**
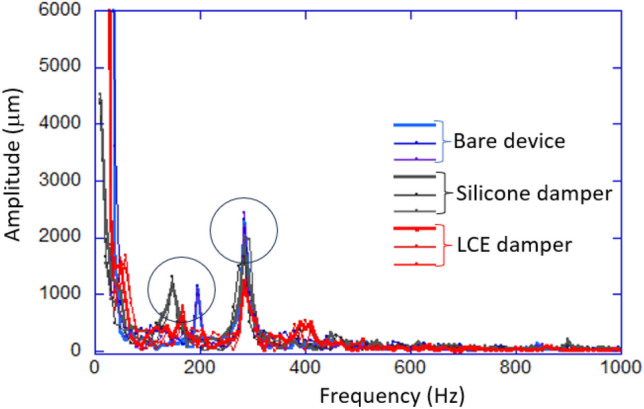
Illustration of the typical spectra of device frame vibrations, broken into three groups of curves: for the bare device, and with the inserted silicone and LCE damper layers, respectively. The principal device frequency of 10.8 Hz is accompanied by two main resonances: at ca.150 Hz and at 283 Hz, which we select for analysis.

The measurement of vibration with the laser Doppler vibrometer is standard: we record a time-series of position values (calculated from the Doppler shift data the instrument obtains) over a specific period of time. The time resolution of 0.2 ms turned to be sufficient to resolve a kHz frequency range in the spectrum, while we found that extending the recording period past a certain optimal time does not improve the low-frequency resolution of the spectrum. After FFT, the typical spectra of device vibration took the form illustrated in Fig. 5. We select two key resonances of the device, as marked in the plot, and analyze them from two aspects: the height of the resonance peak, and the *Q*-factor. It is clear that these normal modes have a different nature: the ca.150 Hz resonance shifts its natural frequency to much lower values with the addition of a damper elastomer layer, while the 283 Hz resonance has its natural frequency evidently not affected by the damper. We, therefore, assume that the first of these resonances is less representative of the casing vibration, since it is so affected by the small added weight of an elastomer, while the second (main) normal vibration mode at 283 Hz is our main focus.

Each material underwent 20–30 independent experiments, with each experiment comprising 10,000 data points for analysis. After removing anomalies potentially caused by background noise, the average values and error analysis are presented in Table 2, which gives the summary of damping of these main resonances in our device. It allows comparison of the test parameters (the resonance peak amplitude, and the *Q*-factor) across many independent measurements. It is clear, both from the graphic in Fig. 5 and the Table 2 that in our device setup we are not achieving the optimal use of damping elastomer. Comparing with the results on *Q*-factor in Table 1, the LCE does not achieve the desired overdamping regime. Nevertheless, in the same conditions, the suppression of the resonant vibration by LCE is clearly more efficient than by the silicone elastomer, which is the main point of this paper. For a proper study of device vibration suppression, one has to work closer with the manufacturer and identify the optimal locations and configuration of the damper insertion, which was not in the scope of our work here. Such an optimization of engineering design requires a lot of trial and error, and is completely device-specific. The general principles of constrained layer damping have to be observed, but even our particular device (the drill) has too many options of where to place the damper pads for us to explore this aspect.

**Table 2:** Comparison of peak amplitudes (marked A:) and *Q*-factors (marked Q:) for the two resonances, calculated from the data presented in Fig. 5.

	A: Bare	A: Si	A: LCE	Q: Bare	Q: Si	Q: LCE
@150Hz	1100 ± 10	1220 ± 50	650 ± 57	15 ± 2	7 ± 2	8 ± 3
@283Hz	2320 ± 50	1800 ± 140	1120 ± 50	16 ± 4	13 ± 2	9 ± 3

## Conclusions

In the field of vibration and acoustics, there is little awareness of this remarkable new material that shows overdamping (which means $Q <1$ or $\tan \delta >1$) at frequencies in the 100–1000 Hz range. This paper attempts to bring these fields closer together, by doing an ‘imperfect job’ of vibration testing in just a few characteristic settings and using only the mainstream LCE not optimized for high-frequency damping (and also not embarking on a wider exploration and comparison of different damping materials). Our aim has been to point out that vibration damping in LCE is not related to any kind of soft elasticity, let the reader understand that the underlying theoretical mechanisms are not clear, and demonstrate that LCE offers a very good damping. Indeed, the results demonstrate the superior LCE performance in constrained layer damping geometry, compared to the standard materials currently used in industry.

Since there is a direct relationship between the *Q*-factor describing the attenuation of a resonance, and the loss factor $\tan \delta$ describing the dissipation in a linear dynamic-mechanical test, and many previous studies have already pointed at the anomalously high $\tan \delta$ in nematic LCE^2,3,7^, we did expect to find that LCE outperforms other damping materials in our vibration tests. More detailed studies, especially with the damping in specific devices, need to be conducted to bring this concept to the practical application domain.

On the fundamental level, there are two topics remaining open and waiting for research. There is a need of consistent theoretical understanding of high-frequenmcy dissipation mechanism in LCE. And there is a need to optimize the LCE materials for this use: the mainstream thiol-acrylate main-chain LCE that we used as a simple example is certainly not the optimal damper because the rotational motion of mesogens is restricted in the connected main chain. For instance, the side-chain nematic LCE used to show a much higher $\tan \delta$^2,6^, and so would be expected to have a stronger attenuation. All of these avenues remain attractive for future research and development.

## Methods

This section gives some detail on the damping materials used, and the design of two experiments reported.

### Materials

The elastomers involved in this study are deliberately ‘standard’: optimizing the material damping was not our aim here–instead we compared distinct classes of damper materials. The classical elastomer is polydimethylsiloxane (PDMS), from the Sylgard 189 kit supplied by Dow Corning. We compose this elastomer with 10% hardener, achieving the soft transparent rubber material with Shore A index = 68 and casted 2 mm thick plates from it.

The main-chain LCE material has become the mainstream material of choice after the original papers have developed this concept of thiol-acrylate chains^23,24^. The commercially available diacrylate mesogens RM257 (CAS 174063-87-7, described in detail in the cited papers) have been purchased from Daken Chemicals. The dithiol flexible spacers (CAS 14970-87-7, EDDT) and the four-functional thiol crosslinkers (CAS 7575-23-7, PETMP) were purchased from Merck-Sigma-Aldrich. We composed this elastomer with 10% crosslinker density, achieving a soft white (nematic polydomain) rubber material and casted 2 mm thick plates from it. The Shore A index = 65 was measured, although recent studies have pointed out that due to the local polydomain-monodomain transition in this ‘Hertz indentation’ geometry, the Shore A value could be relaxing to much lower values over a long time^25^.

The 2 mm thick Sorbothane shock and vibration damping plates were purchased from Sorbothane, Inc in Kent, Ohio, and used as received.

### Dynamic vibrometer

To form the uniform damping layer in constrained geometry, we used the standard steel rulers (25 mm wide, 30 cm long) and made a Teflon mould for curing the polymer (silicone or LCE) in exactly this area, and 2 mm thick. In the case of Sorbothane, the 2 mm-thick plate was supplied directly and cut into this area. After curing, the polymer was super-glued to the steel rulers on each side, and mounted in a heavy vice, as Fig. 1 illustrates.

The plate resonance study was designed utilizing the PCB Piezotronics 288d01 as force actuator and the PCB 208c05 force accelerometer as the detector (see Fig. 1), both controlled by the 4-channel Digilent AnalogPro 3000 analyzer, model ADP3250. The steel rulers, on their own or bonded with a 2 mm damping layer in between, were fixed in a heavy vice, the actuator and the accelerometer detector superglued at the specified positions, and the “frequency-sweep”signal was supplied to the actuator. For quality measurement, and the successful spectrum transformation, the parameters of frequency sweep need to be carefully selected to optimize the outcome of FFT. In our case, we were sweeping from 100 Hz to 20 kHz in 5 seconds, at a sample rate of 3000 Hz, collecting the data for 15 seconds. The FFT process native to Digilent AnalogPro was run on every time sequence to produce the spectra as shown in Fig. 2.

**Fig. 6:**
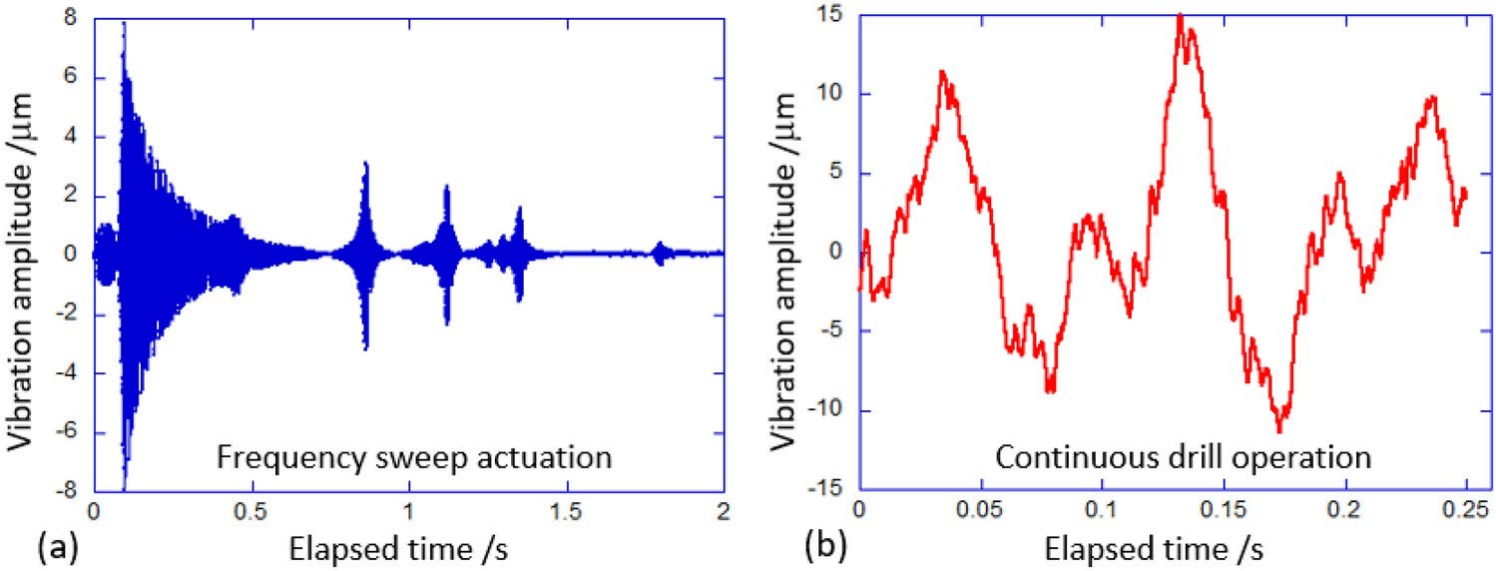
Illustration of the typical raw data from the two tests: (**a**) The spot vibration amplitude in response to the frequency sweep actuation of the vibrating plate, and (**b**) the spot vibration amplitude of the drill casing during its load-free operation.

### Lased Doppler vibrometer

For this study, we used a commercial handheld cordless drill. To insert an added damping layer (either silicone or LCE), we had to open the drill case to expose the motor and its surrounding space. We then lowly introduced the viscous pre-cured polymer mix into this space until the polymer slightly overflows when the case is closed, ensuring full and even coverage inside the gap between the motor and the casing. Allowing the polymer to cure, we then carefully removed any excess polymer and cleared the ventilation areas, before closing the casing, as illustrated in Fig. 4.

The device resonance study was designed utilizing the Optomet Laser Doppler Vibrometer, Vector-Start model. Its standard operation is to pick up a perpendicular beam reflection from a surface spot (see Fig. 4) and, via the Doppler shift, measure its velocity along the surface normal. From this, the surface position (height) is deduced as function of time. The vibrometer data was fed directly into the 4-channel Digilent AnalogPro 3000 analyzer (ADP3250) for analysis. For quality measurement, and the successful spectrum transformation, the time resolution and the recording duration need to be carefully selected to optimize the outcome of FFT. In our case we were recording for 0.25 seconds with resolution of 0.025 millisecond. Figure 6 shows an example raw signal in both tests, to illustrate the nature of the measurement and the demand on FFT analysis.

## Data Availability

All data generated or analysed during this study are included in this published article, or available from the authors on request.
